# Safe Prescribing: A Quality Improvement Initiative Promoting Anti-Cholinergic Burden Scoring and Medication Review in a Community Mental Health Team for Older People

**DOI:** 10.1192/bjo.2026.11467

**Published:** 2026-06-30

**Authors:** Mark Rafferty, Ornaith Quinlan, Lina Zarouf-Jesri

**Affiliations:** Louth Meath Mental Health Services, Drogheda, Ireland

## Abstract

**Aims:**

By January 2026, 90% of eligible patients will have a documented anticholinergic burden (ACB) score enabling identification of at-risk service users and medication risk management through shared decision-making and partnership working with service users and their general practitioners.

**Methods:**

A structured QI framework was applied, incorporating process mapping, a driver diagram, and iterative Plan–Do–Study–Act (PDSA) cycles. Process mapping identified gaps in assessment, documentation and communication pathways. Stakeholder engagement included MDT discussions, an inpatient pharmacist's guidance on ACB scoring resources, and feedback from community GPs.

PDSA cycles were implemented:

1. MDT Education and updated new patient assessment proforma: MDT teaching on anticholinergic burden and embedding an ACB scoring prompt and ACBcalc.com link into the new patient assessment proforma.

2. Patient/carer involvement: development of a written information leaflet for patients with high burden (ACB ≥3).

3. Eligibility refinement: focusing ACB scoring on patients with cognitive impairment, falls risk, or polypharmacy (≥5 medicines).

4. Enhanced communication: integrating ACB scoring and optimisation recommendations into GPcorrespondence and MDT discussions.

5. Sustainability: monthly run charts, repeated audit cycles, and ongoing MDT feedback.

Process measures included ACB documentation rates, communication of ACB scores to GPs, and documentation of optimisation actions. Outcome measures examined the percentage of eligible patients with a documented ACB score and the percentage of patients with high ACB (≥3) who had a documented optimisation plan. Balancing measures assessed the time impact and clinician workload associated with implementing routine ACB scoring.

**Results:**

ACB scoring increased to 100% of eligible patients by December 2025, with a sustained upward trend across the 6-month study period. Overall, 66% of eligible patients (23/35) had documented ACB scores, reflecting early implementation months. All high-burden patients (8/8) had documented optimisation plans.

Process measures showed that 96% of completed scores (22/23) were communicated to GPs, and 100% of high-ACB patients (score ≥3) had shared decision-making discussions.

Balancing measures indicated improved efficiency following introduction of the quick-reference guide, ACBcalc.com link, and refined eligibility criteria.

**Conclusion:**

Routine ACB scoring was successfully embedded into MHSOP practice, improving identification of high-risk patients, deprescribing opportunities and communication with GPs. The intervention is low-cost, sustainable and reproducible, with potential to reduce medication-related harm in older adults.

